# CBF transcription factors involved in the cold response of *Camellia japonica* (Naidong)

**DOI:** 10.7717/peerj.12155

**Published:** 2021-09-09

**Authors:** Menglong Fan, Rui Zhou, Qinghua Liu, Yingkun Sun

**Affiliations:** College of Landscape Architecture and Forestry, Qingdao Agricultural University, Qingdao, Shandong, China

**Keywords:** CBFs, *C. japonica* (Naidong), PacBio, Low temperature, Winter camellia, Abiotic stress

## Abstract

CBFs belong to the ERF subfamily of the AP2 supergene family and often play an important role in the cold acclimation of temperate plants. However, the role of CBFs in *Camellia japonica* (Naidong), the only *Camellia japonica* population found in the temperate zones of China, remains unclear. It is very important to study the genetic composition of *C. japonica* (Naidong) to adapt to low temperature for Camellia species. Using full-length transcriptome data, we identified four CjCBF genes that respond to cold stress and analyzed their evolutionary relationships, domains, and expression patterns. The phylogeny of CBFs of 19 angiosperms divided the genes into three categories, and the four CjCBFs belong to a small subcluster. The strong response of *CjCBF1* to cold treatment and its sustained high level of expression indicated that it plays an important role in the process of cold acclimation. A yeast two-hybrid assay revealed an interaction between *CjCBF1*, *CjCBF2*, and *CjCBF5*, and subcellular localization confirmed this finding. The expression of CjCBFs was tissue-specific: CBF1 was mainly expressed in leaves, and CBF3 was mainly expressed in stem. The responses of the four CjCBFs to drought and high temperature and the effect of light were also characterized. Our study provides new insight into the role of CBFs in the cold response in *C. japonica* (Naidong).

## Introduction

Low temperature stress is an important abiotic stress that greatly affects the distribution of plants and social and economic development worldwide. The study of cold resistance mechanisms is becoming increasingly important as threats to the ecological environment grow. Plants can reduce the damage caused by low temperature through complex mechanisms, such as physiological metabolic pathways and molecular regulation (Agarwal et al., 2006a). Temperate plants can improve their cold tolerance by experiencing low, non-freezing temperatures (*i.e.,* cold acclimation). This process involves stress perception, signal transduction, and the regulation of protein-coding genes, in which transcription factors act as important regulatory hubs that control a large number of genes to mediate the response to different stresses.

C-repeat binding factors (CBFs) belong to the ERF family of the *AP2* supergene family, which has a common DNA-binding domain and two conserved motifs. They are distinguished by two signature sequences (PKK/RPAGRxKFxETRHP and DSAWR) (Haake et al., 2002). In some plants, CBFs have been shown to be key regulators in the cold acclimation pathway (Stockinger, Gilmour & Thomashow, 1997; Medina et al., 1999; Tayeh et al., 2013; Francia et al., 2016; Würschum et al., 2017) and are specifically associated with the C-repeat/dehydration-responsive motif (CRT/DRE; G/ACCGAC) of the promoter region of the *COR* gene (Lata & Prasad, 2011), which can mobilize a large number of genes involved in cold acclimation independent of the ABA pathway (Kodama et al., 2000; Graham et al., 1997; Ashraf & Foolad, 2007; Kornyeyev et al., 2001). CBF transcription factors have been shown to improve cold resistance in maize, potato, tomato, Arabidopsis, tobacco, and other plants through overexpression verification (Ito et al., 2006; Gilmour et al., 2000; Liu et al., 2011; Dubouzet et al., 2003; Mei et al., 2009; Al-Abed et al., 2007; Tang et al., 2011; Hsieh et al., 2002a; Hsieh et al., 2002b). In addition, previous studies have shown that CBF (DREB) not only participates in low temperature stress but also responds to drought and saline-alkali stress.

However, the overexpression of CBF transcription factors can inhibit plant growth and development and even lead to sterility (Tillett et al., 2012). This problem can be solved by incorporating different promoters into overexpression vectors with *CBF* genes of different species. For example, the incorporation of *Arabidopsis* DREB1A and rd29A into an expression vector was not observed to significantly inhibit growth and sterility (Kasuga et al., 1999). *AtCBF1*, *AtCBF2*, and *AtCBF3* in *A. thaliana* are not only involved in the regulation of cold acclimation but also basic cold tolerance; however, *AtCBF2* appears to be more important than *AtCBF1* and *AtCBF3* in the process of cold acclimation (Zhao et al., 2016). In *Arabidopsis thaliana*, CBF2 negatively regulates CBF1 and CBF3, but CBF1 and CBF3 do not directly regulate the expression of other CBFs; instead, they coordinate the regulation of some downstream target genes (Novillo, Medina & Salinas, 2007). Thus, studying the combined expression of multiple genes is essential for improving plant cold resistance.

*Camellia* plants are mostly distributed in the subtropics and are not tolerant of low temperatures. Temperature greatly affects the economic value of *Camellia* plants. *C. japonica* (Naidong), which is the most northerly distributed *Camellia* species in China, has excellent cold resistance, but molecular research has been limited because of a lack of genomic sequences. Transcriptomic study of *C. japonica* (Naidong) has shown that *α*-jasmonic acid may play an upstream signaling role in the response to cold stress (Li et al., 2016). However, no studies have identified *CBF* genes in *C. japonica* (Naidong) nor have characterized their expression patterns in the response to cold stress.

Here, we identified and isolated four low temperature-responsive CjCBFs transcription factors from low temperature treatment full-length transcriptome data (PacBio). Structure analysis, phylogenetic analysis, Gene Ontology (GO) annotation, and the expression patterns of genes were clarified by bioinformatics technology, and the role of CjCBFs in the response to low temperature stress was characterized in detail. Overall, this study provides valuable information that could be used to improve the cold tolerance of *Camellia* plants.

## Materials and Methods

### Identification of new CjCBFs genes in *C. japonica* (Naidong) involved in cold tolerance

We used our *C. japonica* (Naidong) low temperature and drought full-length transcriptome data based on PacBio and Illumina sequencing (PRJNA689105) as the local database. The protein sequences of CBF family genes (*i.e.,* *AtCBF1*, *AtCBF2*, *AtCBF3*, *AtCBF4*) in Arabidopsis thaliana were queried to search the local databases using the BLASTP method with an *E*-value threshold of <1e−20. Then, HMMER3.0 (Potter et al., 2018) was used to further screen sequences using default parameters (E<1e−5), the AP2 domain was based on Pfam database (Pfam accession, PF00847). The longest protein was selected as a representative for the gene. Sequences that do not contain the complete AP2 domain and signature sequences were eliminated.

### Sequence analysis

ExPASy (Gasteiger et al., 2003) was used to analyze the relative molecular weight, isoelectric point, hydrophilicity, and other basic information of CjCBFs according to default parameters.

### Motif and phylogenetic analysis

MEME (http://meme-suite.org/tools/meme) was used for the conservedmotif analysis of CjCBFs and AtCBFs proteins, with the maximum number of motifs set at ten. The parameters were as follows: minimum motif width, 6; maximum motif width, 50; Selected the motif classic mode and 0-order model of sequences. MAFFT was used for sequence alignment according to default parameters (FASTA format, auto). The R package ggmsa was used to visualize the sequence alignment. In this study, *C. japonica* (Naidong) and 18 other plant species were selected from dicotyledons and monocotyledons, including *Brachypodium distachyon, Oryza sativa* and three additional monocotyledons, as well as *Camellia japonica, Arabidopsis thaliana, Cucumis sativus* and 19 other dicotyledons. The CBF-like protein data of the 19 species (with the exception of *C. japonica*) were downloaded from the NCBI Database. The protein sequences were selected by HMMER3.0. If the AP2 domain was truncated, or the AP2 domain match *E*-value exceeded 1e−5, the protein sequences were excluded. Based on this, 424 CBF-like genes were identified from the 19 plant species in order to construct the phylogeny tree. The phylogeny was constructed using the maximum likelihood method with 1,000 bootstrapping replicates in MEGA (v. 6.0) (Tamura et al., 2013). The R package ggtree (v2.0.4) was used to visualize the phylogenetic tree.

### GO and interaction analysis

KOBAS (Xie et al., 2010) (http://kobas.cbi.pku.edu.cn/) and *A. thaliana* (thale cress) were used for the GO enrichment analysis. The sequences of four *CjCBFs* were inserted separately into pGADT7 Vector and pGBKT7 Vector; pGADT7-T+pGBKT7-Lam was the negative control and pGBKT7-53+pGADT7-T was the positive control. In order to verify self-activation, the pGADT7+ pGBKT7 - *CjCBF1/ CjCBF2/ CjCBF3/ CjCBF5* plasmid was transformed into Y2HGold yeast competent cells, with 30 mg/ml *α*-gal employed to detect the self-activation. Furthermore, in order to determine the suitable concentration of AbA, the inhibitory self-activated AbA concentrations of 0.20 µl/ml, 0.30 µl/ml, 0.50 µl/ml, 0.70 µl/ml and 0.90 µl/ml were adopted for the five working solutions. The pGBKT7 and pGADT7 + CjCBF1, pGBKT7 and pGADT7 + CjCBF2, pGBKT7 and pGADT7 + CjCBF3, and pGBKT7 and pGADT7 + CjCBF5 plasmids were transformed into Y2HGold yeast competent cells, respectively, to exclude false positives. Moreover, the pGADT7+ *CjCBFs* and pGBKT7 + *CjCBFs* plasmid was transformed into Y2HGold yeast competent cells to verify the interaction between CjCBFs.

### RNA-seq data

In order to explore the transcriptional expression of CjCBFs at low temperature stress across time, we evaluated our low temperature stress transcriptome (PRJNA689105) under a 4 °C treatment lasting 0 h, 12 h, 24 h, and 72 h, with four groups of transcriptome data. The expression of the CjCBFs gene was analyzed and the results were visualized by the R package pheatmap. qRT-PCR technology was employed to verify the transcriptome data and the co-expression method was used to predict the potential lncRNA regulating CjCBFs (Li et al., 2015).

### Subcellular localization

Four DNA sequences encoding CBF proteins were inserted into the super1300-GFP overexpression vector, and *Agrobacterium tumefaciens* GV3101 containing super1300-CjCBF1, super1300-CjCBF2, super1300-CjCBF3, and super1300-CjCBF5 was injected into the Abaxial of tobacco (*Nicotiana benthamiana*) leaves (Li et al., 2013). A Leica DM2500-DM2500 LED fluorescence microscope was used to obtain fluorescence information.

### Promoter cloning and Cis-Element analysis

*C. japonica* (Naidong) DNA was extracted *via* the CTAB method (Doyle & Doyle, 1987) to clone the promoter. The *CjCBF1* upstream 5′ UTR sequence was obtained from the Takara Genome Walking Kit (Takara, Japan) following the manufacturer’s instructions. Design of nested PCR specific primers with primer 5.0 (Table S7). The obtained sequence was connected to a T vector for sanger sequencing and the Cis-Elements in the sequence were analyzed based on the PlantCARE database (Lescot et al., 2002).

### Plant materials and low temperature treatment

*C. japonica* (Naidong) seeds were obtained from the Qingdao Botanical Garden (36°05′N, 120°08′E), Qingdao Agricultural University Cooperative, China, in September 2018. The seeds were sown in sand in the Qingdao Agricultural University culture room (20 ± 2 °C) during the winter until the fourth true leaf appeared. Following germination, the plants were transferred into the artificial climate room of the university (36°31′N, 120°39′E) under normal culturing conditions (plastic pots: top/bottom diameter 20/12 cm, height 26 cm; temperature: 25 ± 2 °C; relative humidity: 60%; soil: peat soil 80% + river sand 20%; natural light) for 3 months.

For the tissue specific expression experiment, we selected seedlings of a similar growth status and divided them into two groups, with three plants per group. Each group was treated at 4 °C for either 0 h or 24 h in a low temperature incubator. Tender roots, stems, leaves, buds, and flowers were collected at 0 h and 24 h. For the heat, cold and light treatments, seedlings were separated into several groups. The first group was placed at 40 °C (from 25 °C to 40 °C with a 1 °C increment per hour) and maintained for 24 h (2000lx light intensity and 60% humidity). The second group was placed at 40 °C (from 25 °C to 40 °C with a 1 °C increment per hour) and maintained for 24 h (60% humidity). The plants were wrapped with foil to avoid light. The third group was placed at 4 °C (from 25 °C to 40 °C with a 1 °C increment per hour) and maintained for 24 h (2000lx light intensity). The fourth group was placed at 4 °C (from 25 °C to 40 ° C with a 1 °C increment per hour) and maintained for 24 h. (1 degree per hour from 25 °C to 4 °C). The plants were wrapped with foil to avoid light. The drought treatment involved stopping watering for 20 days and measuring the relative soil water content to determine the drought degree (Table S8). Unstressed seedlings were used as the control samples (CK). Three biological replicates were collected from the same position at each collection time for RNA isolation.

### RNA extraction and Expression analysis of qRT-PCR

The total RNA extraction was performed using the SPARKeasy A0305 kit (QingDao, China), following the manufacturer’s instructions. Each treatment comprised three replications. The integrity and concentration were detected by 1% agarose gel electrophoresis and a NanoDrop2000 (Thermo Fisher Scientific, Waltham, MA, USA). First-strand cDNA was prepared using the Takara PrimerScriptTMRT reagent kit with gDNA Eraser, and RT-qPCR primers were designed by Primer 5.0 (Table S7). Takara TB Green was used to set up the Premix Ex Taq qGreTli RNaseH Plus for the qRT-PCR experiment. Per the manufacturer’s instructions, *18S* was used as an internal reference gene, and there were three biological replicates and three technical replicates in each group. The relative expression was determined by a three-step method (95 °C for 5 s, 55 °C for [15 s], and 72 °C for 30 s, repeated 40 times) on an Applied Biosystems StepOnePlus. The gene expression level was calculated according to the quantitative Ct method. The R package pwr was used to conduct Student’s *t*-tests. The R packages corrplot (0.84) and circlize (version 0.4.11) were used to conduct correlation analysis.

## Results

### Identification and characterization of CjCBFs

First, we used the transcriptome data of *C. japonica* (Naidong) as the local blast resource bank to obtain 11 candidate genes. Further screening incorrectly predicted CBF genes and redundant sequences, and four CBF transcription factors responsive to low temperature were identified. They were named according to their homology with CBF transcription factors in related plants (*Camellia sinensis*). The nucleotide and amino acid sequences are provided in Table S1. The full lengths of the *CjCBF* s sequences are 1.82 kb, 1.25 kb, 1.42 kb, and 1.35 kb. The CDSs are between 540 and 837 bp, and the number of amino acids are between 179 and 278. The molecular weights of the four cjCBF proteins are 30282.9 kDa, 19449.25 kDa, 26655.03 kDa, and 26526.23 kDa. The isoelectric point ranges from 4.9 to 9.8. Hydrophilicity ranges from 3.02 to 1.8 (Table 1).

**Table 1:** The physicochemical properties for the 4 CBF gene family members in *C. japonica*.

**Gene name**	**Full length (kb)**	**CDS (bp)**	**Number of amino acids**	**Molecular Weight** (**kDa**)	**Theoretical pI**	**Grand average of hydropathicity**	**Aliphatic index**	**Instability index**
*CjCBF1*	1.82	837	278	30282.91	4.92	−0.533	66.08	53.31
*CjCBF2*	1.25	540	179	19449.25	9.18	−0.411	74.19	51.14
*CjCBF3*	1.42	741	246	26655.03	5.25	−0.407	66.71	46.86
*CjCBF4*	1.35	720	239	26526.23	5.50	−0.328	74.35	48.40

### Analysis of conserved motifs

**Figure 1:**
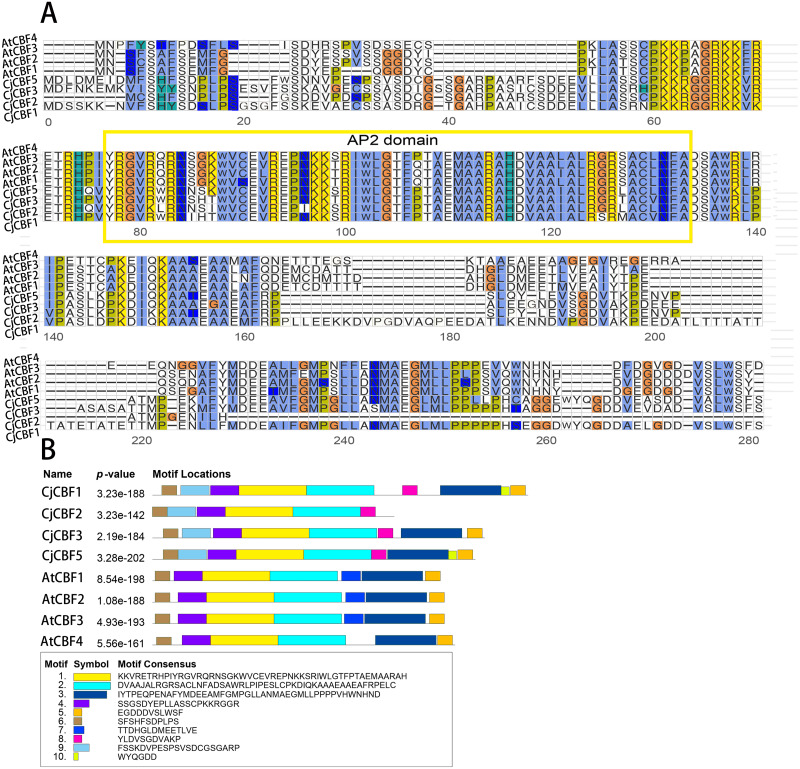
Multiple sequence alignment of CBFs gene and Conservative motif analysis. (A) The AP2 domain is represented in the yellow box. (B) The architecture of the 10 conserved protein motifs predicted *via* MEME analysis. Each motif is represented in a different color (Motif 1–10).

A comparison of the CjCBFs sequences (Fig. S1) revealed that the internal similarity of CjCBFs was 73.96%, and the domain sequence was highly conserved. Comparison of the domain sequences of *A. thaliana* and *C. japonica* (Naidong) (Fig. 1A) showed that the domain of *C. japonica* (Naidong) consists of the AP2 family universal DNA-binding domain (AP2 domain) and two signature sequences (DS-W-L), which were slightly different from the conserved domain of *A. thaliana*. We then analyzed the conserved motifs of *A. thaliana* and *C. japonica* (Naidong) and identified 10 motifs (Fig. 1B) with amino acid lengths between 6 and 50. In particular, Motif10 (WYQGDD) was the shortest (6 residues), while Motif1 (KKVRETRHPIYRGVRQRNSGKWVCEVREPNKKSRIWLGTFPTAEM AARAH) and motif2 (DVAAJALRGRSACLNFADSAWRLPIPESLCPKDIQKAA AEAAEAFRPELC) were the longest (50 residues) and contained the AP2 domain. Only CjCBFs have motif8 (YLDVSGDVAKP), and only *A. thaliana* has motif7 (TTDHGLDMEETLVE). Only CjCBF1 and CjCBF5 have motif10. The functions of most of the motifs have not yet been elucidated. The similarity of motifs within species indicates that CBFs within species are highly conserved. The composition of different conserved motifs may be related to variation in the functional differentiation of genes.

### Analysis of the phylogenetic tree

**Figure 2:**
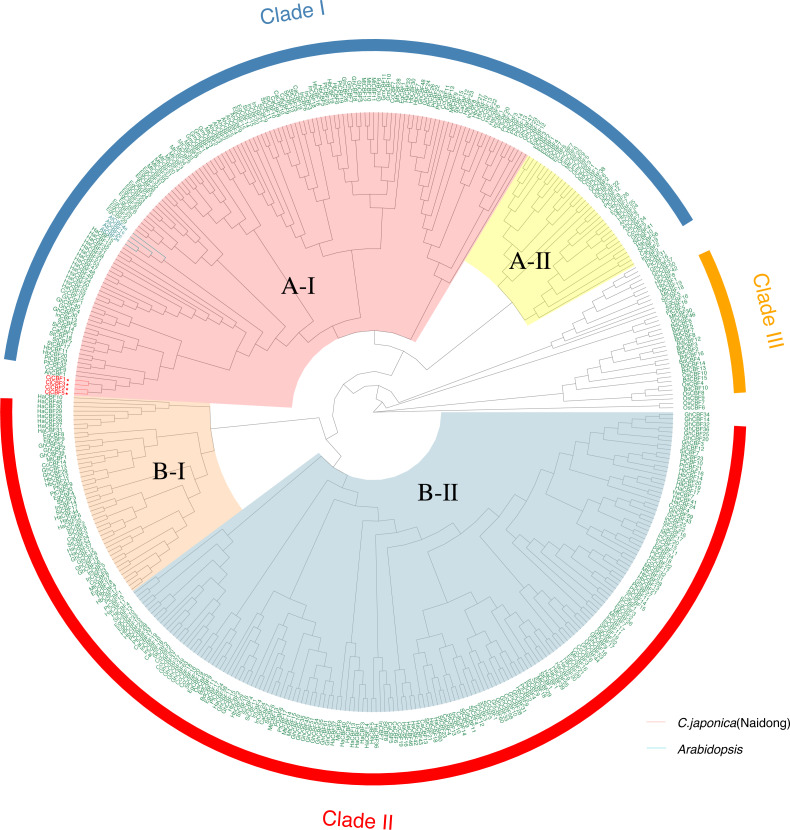
Phylogenetic analysis of CBFs. Maximum Likelihood phylogenetic trees of the protein sequences of 424 genes encoding CBFs from 19 angiosperms. camellia (Cj: *Camellia japonica*) are marked red in the evolutionary tree, and Arabidopsis (At: *Arabidopsis thaliana*) are marked blue in the evolutionary tree. The other species are: Eucalyptus (Eug: *Eucalyptus grandis*); soybean (Gm: *Glycine max*); Cucumber (Cs: *Cucumis sativus*); Chili Peppers (Ca: *Capsicum annuum*); Cotton (Gh: *Gossypium hirsutum*); potato (St: *Solanum tuberosum*); western balsam poplar (Pt: *Populus trichocarpa*); Kiwi fruit (Ac: *Actinidia Chinensis*); Morning glory (Pn: *Pharbitis nil*); Sunflower (Ha: *Helianthus annuus*); Rubber Tree (Hb: *Hevea brasiliensis*); sesame (Si: *Sesamum indicum*); Carrot (Dc: *Daucus carotavar.sativa*); Medicago (Mt: *Medicago truncatula*); Brachypodium distachyon (Bd: *Brachypodium distachyon*); rice (Os: *Oryza sativa*); oil palm (Eg: *Elaeis guineensis*).

To characterize the evolutionary relationships of *CBF* s among species, we constructed a genetic tree (Fig. 2) using 424 *CBFs* from 19 species, including dicotyledons and monocotyledons. These genes were generally divided into three categories. Monocotyledons and dicotyledons were clearly separated, and dicotyledons were divided into two subclasses: Clade I and Clade II. However, some monocotyledons (*e.g.*, oil palm) were divided into A-II subcluster in Clade I, and rice was also partially distributed in this subcluster. This cluster is close to Clade III, with groups containing only Monocotyledons. This may represent the region of functional overlap of dicotyledon and monocotyledon CBFs. This also indicates that this cluster of *CBF* ancestral genes had differentiated before the separation of monocotyledons and dicotyledons and thus that the function and pedigree of *CBFs* became gradually enriched. A-I and B-II were the largest subclusters in Clade I and Clade II, respectively. *CjCBFs* were divided into A-I subclusters and clustered with *A. thaliana*. *CBFs* were highly conserved within species, and *CBFs* of the same species often clustered together.

### GO analysis

**Figure 3:**
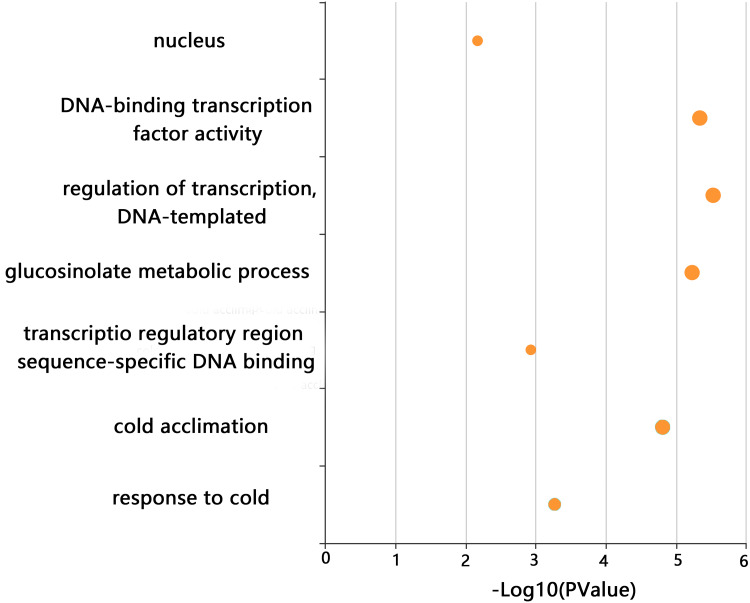
GO analysis. The GO enrichment results of CjCBFs based on KOBAs. The enrichment degree increases with the -log10 (p value) value.

GO enrichment analysis of *CjCBFs* was conducted to better understand the biological function of CjCBFs. CjCBFs were enriched in nucleus (GO:0005634), DNA-binding transcription factor activity (GO:0003700), regulation of transcription, DNA-templated (GO:0006355), transcription regulatory region sequence-specific DNA binding (GO:0000976), glucosinolate metabolic process (GO:0019760), cold acclimation (GO:0009631), and response to cold (GO:0009409). The results of the GO analysis confirmed that CjCBFs play a role in cold acclimation (4 °C) (Fig. 3) (Xie et al., 2010).

### Expression model of *CjCBFs* based on RNA-seq

**Figure 4:**
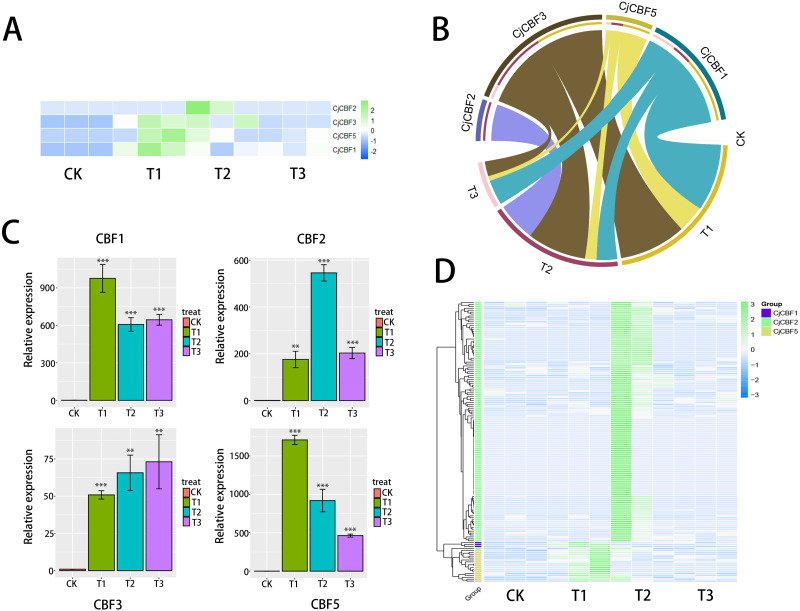
Expression analysis based on the transcript group data. (A) Expression pattern of CjCBFs in response to cold 0 h(CK), 12 h(T1), 24 h(T2), 72 h(T3) treatments, The RNA-seq data were normalized based on the mean expression value (log2fold change, log2FC) of each gene, green and blue boxes indicate high and low expression levels, respectively. (B) Correlation analysis of four CjCBFs with different low temperature treatment groups (cyan region connects *CjCBF1* and four treatment groups, yellow region connects *CjCBF5* and four treatment groups, brown region connects *CjCBF3* and four treatment groups, purple region connects *CjCBF2* and four treatment groups), the wider the region, the higher the correlation. (C) Verification of the CjCBFs expression in the transcriptome *via* qRT-PCR. (D) Differential expression heat map of the lncRNAs that regulate CjCBFs based on the full-length transcriptome data. The data were normalized based on the mean expression value (log2fold change, log2FC) of each lncRNAs, green and blue boxes indicate high and low expression levels, respectively. Yellow, green and blue groups represent the regulation of *CjCBF2*, *CjCBF1*, and *CjCBF5*, respectively.

**Figure 5:**
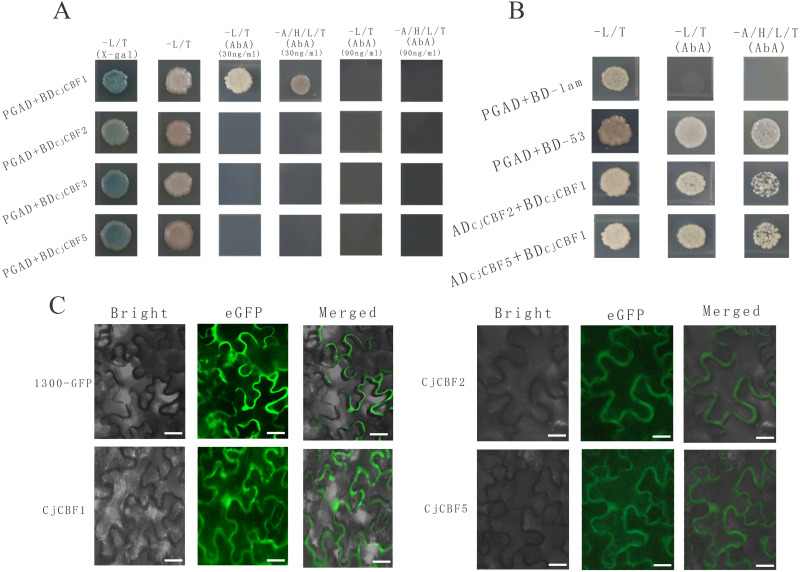
Protein interaction identification and subcellular localization. (A) Self-activation verification. White colony indicates the ability of yeast to grow on the deficient medium, blue color indicates that the self-activating activity of CjCBFs was not able to grow on the deficient medium supplemented with AbA. This points towards the inhibition of the self-activating activity. (B) Yeast two-hybrid. Yeast cells exhibit normal growth on the nutrient-deficient medium, indicating the interaction between CjCBF1, CjCBF2 and CjCBF5. (C) Subcellular localization of Super1300, CjCBF1, CjCBF2 and CjCBF5 in Nicotiana benthamiana. In bright, the black line is the outline of the cell. For eGFP, the green region is the expression site of the plasmid with GFP fluorescent protein and the CjCBFs gene.

Based on the data analysis of the full-length transcriptome under treatment with low temperature stress, the standardized FPKM cluster analysis heat map (Fig. 4A) shows that *CjCBFs* responded strongly to low temperature. Although *CjCBF1* played a role in all three gradient treatment groups, it was significantly up-regulated at the 12 h stage, and the strength of the effect was significantly higher in the 12 h stage than in the 24 h and 72 h stages. The response of *CjCBF3* to low temperature was basically the same in the three treatment groups. *CjCBF2* responded in all three treatment groups but was significantly up-regulated in 24 h and then decreased in 72 h. The expression of *CjCBF3* and *CjCBF5* was similar to that of *CjCBF1*, except that *CjCBF5* was significantly up-regulated in 24 h but then decreased significantly and remained expressed at a low level. qRT-PCR was used to verify the relative expression of *CjCBFs*. The pattern was nearly identical, indicating that our transcriptome results were reliable (Fig. 5C). The correlation analysis also supports this finding (Fig. 4B). There is much evidence showing that lncRNA participates in biological processes by regulating gene expression, including responses to stress in plants. Our SMART and next-generation sequencing data were used to predict possible lncRNA transcripts. A total of 147 possible *CjCBF*-containing transcripts (Fig. 4D) were predicted by lncRNA.

### Interactions of CjCBFs

To further study the post-transcriptional regulation mechanism of CjCBFs under cold stress, we characterized the interactions among CjCBF1, CjCBF2, CjCBF3, and CjCBF5 using the Y2Hgold yeast two-hybrid technique. All CjCBFs were completely inhibited when the concentration of Aureobasidin A (AbA) reached 90 ng/µl, while the corresponding AbA concentration for CjCBF2, CjCBF3, and CjCBF5 was 30 ng/µl (Fig. 5A), The results of self-activation verification showed that the self-activation ability of CjCBF1 was significantly higher than that of other CjCBFs, CjCBF2-PGAD, CjCBF3-PGAD, and CjCBF5-PGAD were transformed into yeast Y2HGold cells in CjCBF1-PGBD; PGAD-T and PGBD-lam were transformed into the negative control, and PGBD-T and PGBD-53 were transformed into the positive control. The results of interaction verification showed that the yeast cells co-transformed with CjCBF1 and CjCBF2, CjCBF1 and CjCBF5 could grow normally on the nutrient-deficient medium, indicating that CjCBF1 could interact with CjCBF2 and CjCBF5 (Fig. 5B). To verify the yeast two-hybrid results, we analyzed the subcellular localization of CjCBF1, CjCBF2, and CjCBF5. CjCBF1 and CjCBF5 were located in the nucleus and cell membrane, and CjCBF2 was located in the cell membrane (Fig. 5C). These results were consistent with the yeast two-hybrid results. Several *CjCBFs* were expressed to aid the ability of *Camellia japonica* to cope with low temperature stress.

### Expression of CjCBF*s* in five *Camellia japonica* tissues

**Figure 6:**
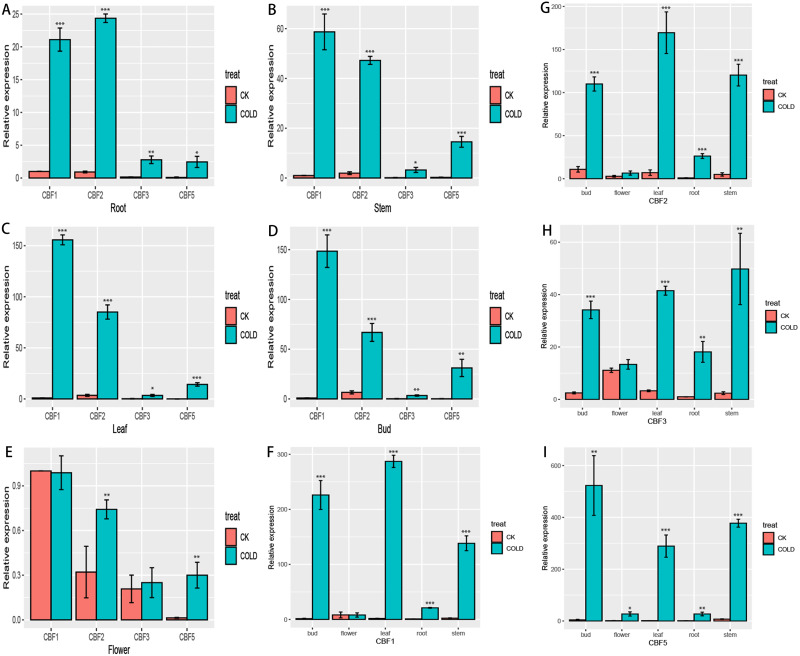
Organ-specific expression of four *CjCBFs*. (A–E) We measured the relative expression amounts of four CjCBFs in each tissue type for A, B, C, D, E, and set the expression amounts of *CjCBF1* in the CK group to one for each tissue. This was then used to calculate the relative expression amounts of other CjCBFs for all tissue types. (F–I) We measured the relative expression of each CjCBFs in five tissues got F, G, H, I, and set the expression of each CjCBFs in the root of the CK group to 1. This was then used to calculate the relative expression of each CjCBFs in other tissues.

To more fully characterize the spatial expression characteristics of *CjCBFs* in *C. japonica* (Naidong), the leaves, stems, flowers, buds, and roots of *C. japonica* (Naidong) were treated with 4 °C for 24 h. The expression of *CjCBFs* was detected by qRT-PCR. the results showed, In the normal cultured CK group, the expression of 4 CjCBFs was lower, but it was worth noting that the expression of *CjCBF1*, *CjCBF3* in flowers was higher than that of *CjCBF2*, *CjCBF5*. This suggests that *CjCBF1*, *CjCBF3* may be involved in flower development. after 24 h of 4 °C treatment, 4 CjCBFs were Significant difference, but only *CjCBF1* was highly expressed in all five tissues, especially in the petals, and *CjCBF2*, *CjCBF3*, and *CjCBF5* were expressed at low levels. The expression of *CjCBF2* in roots and flowers was low, and the expression of *CjCBF3* and *CjCBF5* in stems, leaves, and buds was significantly higher, and the expression of *CjCBF5* in buds was higher (Fig. 6). These results suggest that *CjCBF1* may play an important role in the response to cold stress in *C. japonica* (Naidong).

### Bioinformatics Analysis of *CjCBF1* Cis-Elements

**Figure 7:**
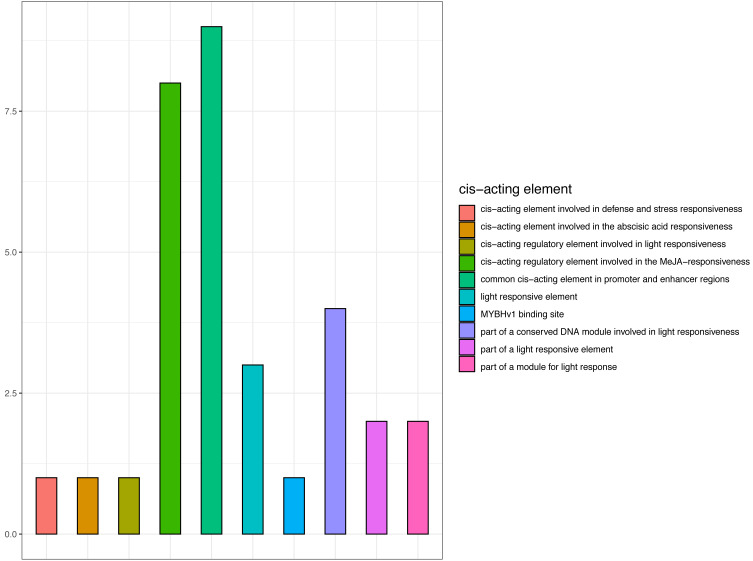
Prediction of cis-elements in the *CjCBF1* promoters.

Promoter cis-elements function as transcription factor binding sites and are key for the transcriptional regulation in response to abiotic stress (Moore et al., 2010). As *CjCBF1* plays an important role in CjCBFs, we cloned and analyzed the 1,400 bp upstream sequences of *CjCBF1*. Bioinformatics analysis revealed the sequence to contain 31 cis-acting elements (Fig. 7 and Table S3), including 12 photosensitive responses and related cis-elements, CGTCA and TGACG motifs (involved in methyl jasmonate responsiveness). The sequence was also rich in TC-rich repeats related to stress, and MYB and MYC related cis elements. The results suggest that *CjCBF1* has a wide response to abiotic stress and that light may play an important role in the expression of CjCBFs.

### Expression of CjCBF*s* under different stresses and the effect of light

**Figure 8:**
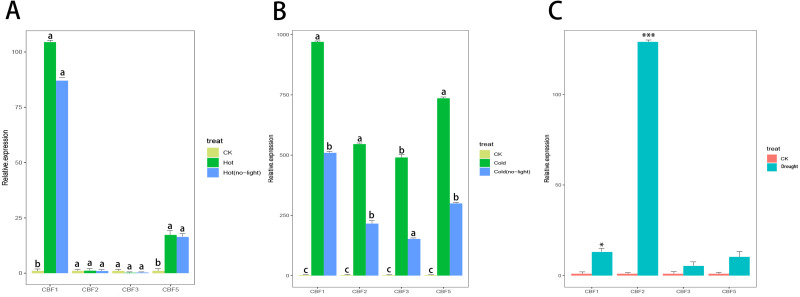
Response of *CBFs* to multiple stresses. (A) Relative expression of CjCBFs under the heat and hot (no-light) treatments. The expression of CjCBFs in each CK group was set to 1. (B) Relative expression of CjCBFs under the cold and cold (no-light) treatments. The expression of CjCBFs in each CK group was set to 1. (C) Relative expression of CjCBFs in the drought treatment. The expression of CjCBFs in each CK group was set to 1.

*Camellia japonica* (Naidong) was treated with high temperature stress, drought stress, low temperature stress, and light stress. Only *CjCBF1* and *CjCBF5* responded to high temperature (40 °C) stress (Fig. 8A), but the expression of *CjCBF1* was significantly lower under 40 °C treatment than under 4 °C for 24 h and decreased slightly under joint high temperature and light stress treatment. Under low temperature and shading stress (Fig. 8B), the expression of *CjCBFs* decreased significantly compared with the non-shading treatment, which indicated that light may not represent a signal that *CjCBFs* respond to at high temperature; however, the effect of light on *CjCBFs* was more important at low temperature. The responses of *CjCBF1*, *CjCBF3*, and *CjCBF5* to drought stress were weak; only *CjCBF2* showed a significant response to drought stress, indicating that the responses of *CjCBFs* in *Camellia japonica* to drought stress are not strong (Fig. 8C).

## Discussion

When plants are subjected to abiotic stress, complex transcriptional and metabolic mechanisms are mobilized. Previous studies have shown that CBF transcription factors are essential for regulating responses to various stresses, and they can mobilize 35% of *COR* genes involved in cold acclimation independent of the ABA pathway. The ICE-CBF-COR pathway is widely known to be one of the core pathways in the response to low temperature stress in many plants, including *A. thaliana*. *ICE1* and its homologous gene *ICE2* dynamically regulate the expression of *CBFs* to ensure that they are expressed at appropriate levels in different periods. However, the regulation of *CBFs* is complex; for example, the promoter has at least one CRT/DRE motif *COR* gene, PHYTOCHROME-INTERACTING FACTOR4 (PIF4) and PIF7 (Barrero-Gil & Salinas, 2018) and the brassinosteroid signaling transcription factors BRASSIN AZOLE RESISTANT1 (BZR1) (Eremina, Rozhon & Poppenberger, 2016). In addition, the number of *CBFs* is often related to the size of the genome (Wang et al., 2019). Five CBF transcription factors have been identified in *Camellia sinensis* genomes, but the response patterns and characteristics of CBFs in *C. japonica* (Naidong) remain unclear. As the northernmost *Camellia* in China, exploration of the cold resistance mechanism of *C. japonica* (Naidong) is particularly important for enhancing the cold tolerance of *Camellia*. However, its genome was not sequenced. Using data from the PacBio and the Illumina full-length transcriptome, four *CjCBFs* were identified. These *CjCBFs* participated in the response to cold stress at non-freezing temperatures, and their expression was highest in the early stage of the stress response. They also responded to high temperature stress and drought stress.

Plants have evolved several strategies to cope with environmental stress. The evolution of multiple copies of genes can permit plants to quickly respond to stress. Multiple copies of *CBF* genes have often been observed (Zhao et al., 2016). However, there are no reference genome sequences of *C. japonica* (Naidong), precluding the possibility of determining the actual origin of these gene copies. In our transcriptome data, we detected multiple CBF transcripts, which have almost the same CDS region and significantly different UTR regions compared with ontological annotations. However, the expression levels of these copies were not the same. We speculate that the UTR region may also play an important role in the CBF pathway of cold-resistant *Camellia japonica*. Comparison with the database revealed that lncRNAs are not predicted in the UTR region of CjCBF; however, this does not mean that the UTR region is not involved in the response to low temperature stress. In the phylogenetic tree, *CjCBF1* and *CjCBF3* are clustered together, and *CjCBF2* and *CjCBF5* are clustered together. This, combined with the results of our yeast experiment, indicates that *CjCBF1*, *CjCBF2*, and *CjCBF5* show different patterns of expression and are regulated differently. *CjCBF3* appears to be a functional redundancy of *CjCBF1* in the response to low temperature stress and does not directly participate in the internal interaction. These results confirm the core role of *CjCBF1* in the response to low temperature stress. Several studies have shown that *CBFs* are not only involved in the stress response but are also involved in the inhibition of growth and development and delayed flowering. Analysis of the expression in five *Camellia* tissues in the absence of cold treatment revealed that *CjCBF1* was highly expressed in flowers, but there was no significant change after cold treatment. Although *CBF* plays an important role in stress, *CBF* overexpression inhibits plant growth (Li et al., 2016; Zhao et al., 2016; Gilmour, Fowler & Thomashow, 2004); there is thus a need to find ways to avoid the negative effects of overexpression of these genes.

We obtained the upstream 1,400-bp sequence of *CjCBF1* by the walking method (Table S2) and identified 12 homeopathic elements related to light, 1 cis-acting element involved in defense and stress responsiveness, and 8 cis-acting regulatory elements involved in MeJA responsiveness (Table S3). There are several light-response homeopathic elements in CjCBFs; We analyzed the response of *CjCBFs* to light under stress in detail through a light control experiment under low temperature. We found that the expression of *CjCBFs* decreased significantly under shading, indicating that light promotes the low temperature response of *Camellia japonica*.

However, light is not a necessary condition. Thus, the study of broad-spectrum and sensitive stress genes is particularly important and is the reason for increased interest in studying the effects of multiple types of stress. This suggests that the expression of *CjCBFs* is indeed different between day and night in winter. Interestingly (Gilmour, Fowler & Thomashow, 2004), our light control experiment under high temperature stress revealed that light did not cause changes in the expression of *CjCBFs*, and light intervention did not affect the response of *CjCBF1* and *CjCBF5* to heat stress. We found that low temperature induces the strongest and most complex regulatory response of *CjCBFs*. In addition, there are MeJA signal elements in the promoter sequence of *CjCBF1*, indicating that it may be regulated by jasmonic acid and may be involved in the metabolism of glucosinolates. *CBF* overexpression in *A. thaliana* promotes the accumulation of glucose, sucrose, and proline, which aids the survival of plants at low temperatures.

*CBFs* are structurally expressed in *A. thaliana*. *CBF1* and *CBF3* are the main components underlying the response to cold stress in plants (Gilmour et al., 2000; Harmer et al., 2000). *CBF2* is involved in the regulation of *CBF3* and *CBF1*. We verified the relationship between *CjCBFs*. *CjCBF1* plays a central regulatory role. *CjCBF1* may be expressed first and then regulate *CjCBF5* and *CjCBF2*. The expression patterns indicated that the three *CjCBFs* were maintained in a dynamic balance, and the rapid up-regulation of *CjCBF1* caused by low temperature breaks this balance. *CjCBF2* and *CjCBF5* negatively regulate *CjCBF1* to maintain its level of expression within a specific range.

## Conclusion

Four CjCBFs involved in the cold response of *C. japonica* (Naidong) were studied. An analysis of the motifs and evolution of these proteins provided information that could be useful for studying other *Camellia* species. The interaction mode of *CjCBF1*, *CjCBF2*, and *CjCBF5* provided new insight into the biological function of CBF transcription factors in *Camellia*. The exploration of various stresses and responses to light expanded the known functions of CjCBF transcription factors and will aid future studies of the mechanism of stress adaptation in *Camellia* and other plants.

## Supplemental Information

10.7717/peerj.12155/supp-1 Supplemental Information 1 Sequence similarity analysis of CjCBFs The white indicates that the structure of this position is not conservative. The color background indicates a conservative structure of the position.

10.7717/peerj.12155/supp-2 Supplemental Information 2 Coding sequence and protein sequence of CBF members from camellia japonica(Naidong) and Arabidopsis

10.7717/peerj.12155/supp-3 Supplemental Information 3 Promoter sequence of CBF1 from camellia japonica(Naidong)

10.7717/peerj.12155/supp-4 Supplemental Information 4 The analysis of cis-elements of promoter

10.7717/peerj.12155/supp-5 Supplemental Information 5 Raw data for Fig. 5C

10.7717/peerj.12155/supp-6 Supplemental Information 6 Raw data for Fig. 7

10.7717/peerj.12155/supp-7 Supplemental Information 7 Raw data for Fig. 8

10.7717/peerj.12155/supp-8 Supplemental Information 8 The primer of qRT-PCR and promoter clon

10.7717/peerj.12155/supp-9 Supplemental Information 9 The relative water content of soil

## Additional Information and Declarations

### Data Availability

The following information was supplied regarding data availability: The data is available at NCBI GEO: PRJNA689105. The raw measurements are available in the Supplemental Files.
