# Supplementary figures and images for: CBF transcription factors involved in the cold response of Camellia japonica (Naidong)
Source: PeerJ. 2021 Sep 9;9:e12155. doi: 10.7717/peerj.12155 (PMC8435204; doi:10.7717/peerj.12155)

843

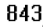

194

394

594

794  
E 40836  
F40

Supplement: Supplemental Information 1 — The white indicates that the structure of this position is not conservative. The color background indicates a conservative structure of the position. [file peerj-09-12155-s001.pdf]
